# Supplementary material for: Adaptive volumetric light and atmospheric scattering
Source: PLoS One. 2020 Nov 18;15(11):e0242265. doi: 10.1371/journal.pone.0242265 (PMC7673549; doi:10.1371/journal.pone.0242265)
Supplement: S1 Appendix — (PDF) [file pone.0242265.s001.pdf]

Basic Principles

Currently, Rayleigh scattering and Mie scattering are used to calculate the scattering coefficient in this section. The difference between the two scattering lies in the type of the particles: air molecules and aerosols. Scattering by air molecules is described by the Rayleigh theory which is considerably wavelength-dependent and isotropic. While scattering by aerosols is expressed as Mie scattering.

0.1 Rayleigh scattering

An important property of Rayleigh scattering is that its phase equation is inversely proportional to the fourth power of the wavelength. And short wavelengths have a stronger scattering intensity than that of the long wavelengths which lead to blue sky in the daytime and orange in the evening. The scattering coefficient of Rayleigh scattering can be described by the following formula :

$$\beta_{\mathrm{R}}(\theta) = \frac{2\pi^2 {(n^2-1)}^2}{3N\lambda^4}\, p_R(\theta) \tag{1}$$

Where θ is the angle between the sight and the light, N is the density of atmospheric molecules, n is the atmospheric refractive index, λ is the wavelength of the incident light, and $p_R(\theta)$is the unitized phase function.

0.2 Mie scattering

Scattering by aerosols is called Mie scattering where Cornette-Shanks function is commonly used as an approximation to the phase function as follows :

$$\beta_{\mathrm{M}}(\theta) = \frac{1}{4\pi}\frac{3(1-g^2)}{2(2+g^2)}\frac{1+\cos^2\theta}{{(1+g^2-2g\cos\theta)}^{3/2}} \tag{2}$$

Where $\beta_{\mathrm{M}}(\theta)$ is the scattering coefficient, θ is the angle between the light direction and the view direction, g is the symmetry of scattering.

0.3 Light Scattering Integral

Light interacts with particles in two ways: Scattering and absorption. Scattering changes the direction of light. While Absorbs converts light into other forms of energy. The light that eventually reaches the camera is observed by the human eye which is divided into two parts: the reflected light of the attenuated object and the internally scattered light (as shown in the formula).

$$L_{Camera} = L_{Object} \cdot e^{-T(O\to C)} + L_{Inner} \tag{3}$$

Where L_{Camera} is the total light intensity finally reaching the camera, L_{Object} is the initial reflected light reflected by the object to the camera, $e^{-T(O\to C)}$ is the attenuation coefficient of reflected light from the object position O to the camera position C, and T is the optical depth.

As shown in Fig. 8, P is defined as a point on the view ray starting at camera location C and terminating at point O. The optical depth $T(A \to P)$ means integral of

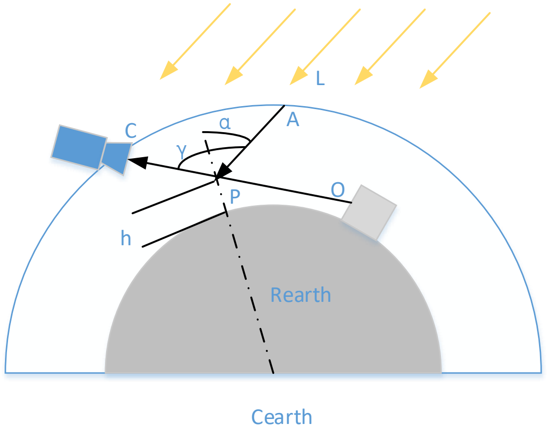

Fig 1. Scattering in the atmospere

the scattering coefficient over the entire path from point A to point P which is defined follows:

$$T(A \to P) = \int_{A}^{P} (\beta_R^e e^{-h(t)/H_R} + \beta_M^e e^{-h(t)/H_M})dt \tag{4}$$

Rayleigh and Mie scattering happen independently by corresponding coefficient β_R^S andβ_M^S at the sea level which is proportional to the particle density scale factor $e^{-h(s)/H_R}$ or $e^{-h(s)/H_M}$. The fraction of the scattered light accumulated on the view direction is given by phase function $p_R(\theta)$and $p_M(\theta)$ for each type. And the light at P is attenuated along the view ray by factor $e^{-O(A \to C)}$.while V(P) is introduced for visibility test of samples. Total in-scattering along the view ray is given by the following integral:

$$L_{Inner} = \int_{A}^{C} L_{Sun} e^{-T(A(s) \to P(s))} e^{-T(P(s) \to C(s))} (\beta_R^S e^{-h(s)/H_R} p_R(\theta) + \beta_M^S e^{-h(s)/H_M} p_M(\theta)) ds \tag{5}$$
