# Supplementary material for: Adaptive volumetric light and atmospheric scattering
Source: PLoS One. 2020 Nov 18;15(11):e0242265. doi: 10.1371/journal.pone.0242265 (PMC7673549; doi:10.1371/journal.pone.0242265)
Supplement: Attachment — Submitted filename: Rebuttal letter.pdf [file pone.0242265.s006.pdf]

Cover Letter

Dear Editor and reviewers

Thank you very much for giving us an opportunity to revise our manuscript. We appreciate the editor and reviewers very much for their constructive comments and suggestions on our manuscript entitled “Adaptive volumetric light and atmospheric scattering” (ID: PONE-D-20-19144).

We have studied reviewers’ comments carefully. According to the reviewers’ detailed suggestions, we have made a careful revision on the original manuscript. All revised portions are marked in highlight in the revised manuscript which we would like to submit for your kind consideration.

Kind regards.

TanShihan

E-mail: 530916232@qq.com

Rebuttal Letter

Dear Editor and reviewers:

Thank you for your letter and the reviewers' comments on our manuscript entitled "Adaptive volumetric light and atmospheric scattering" (ID: PONE-D-20-19144).

Those comments are very helpful for revising and improving our paper, as well as the important guiding significance to other research. We have studied the comments carefully and made corrections which we hope meet with approval. The main corrections are in the manuscript and the responds to the reviewers' comments are as follows (the replies are highlighted in blue).

Replies to the reviewers' comments:

Reviewer #1:

Q1. The section "Related works" should be incorporated into the Introduction, and the Introduction should then end by making clear the distinction between what was accomplished in the related works and the specific methodology of the current work.

Response：Yes, the description of Related work was incorporated into the Introduction as a subsection by which made the logic more natural and continuous. And we added more details to make clear the distinction between the related works and the specific methodology of the proposed work. We then explained clearly why the related work cannot be adapted to the application of this paper and which parts of the related work were new or reused. Finally, the innovation and contributions of this article were introduced. See the highlighted "Introduction" section, "related works" and "overview" subsection

Q2. Points 7 and 9 on p. 4 are a bit vague, although the meaning becomes clearer once the reader gets to the detailed description of the algorithm.

Response：Yes, we modified the points 7 and 9 on p.4 to explain the process more clearly, as shown below: Point 7: Compute the interaction between the multiple light sources and participating media. Point 9: Perform ray marching on each view ray, and accumulate the transparency of each sample. When the accumulated transparency reaches 1, the traversal is terminated.

Q3. Step b in the subsection "Epipolar sampling" on p. 4 is also vague. Specifically, what constitutes a sample, and what does "are marched" mean? Are the samples values at grid points where calculations are performed? Does their spacing determine the spacial resolution, or are calculations also performed as the samples are propagated between the grid points shown in the figures?

Response：Yes, we rewrite the step b in the subsection "Epipolar sampling" on page five. Without ES method, it is too expensive to perform light scattering for every pixel in real-time. The distribution of samples in the screen space is shown in Fig. 2. The number of computations can be dramatically reduced by epipolar sampling, which smartly distributes expensive ray marching samples along the epipolar lines on the screen. We then interpolate between samples where adequate because light intensity varies smoothly along the epipolar lines. We took advantage of epipolar geometry to improve performance without sacrificing visual quality using fewer number of samples. For each epipolar sample, we cast a ray from the camera through which intensity and in-scattering coefficient of light are accumulated by the discretized sampling points with adaptive step size along the ray (This sentence explains "are marched").

Q4. p. 5: "its direction D_uv" - It seems that D_uv should be a vector with dimensions of length and not simply a direction. Is that correct?

Response：In this paper, D_uv means a vector with dimensions of length. Then the vector is normalized and together with the index, the position of the sampling point can be precisely defined.

Q5. Fig. 6c is not entirely clear, specifically the arrows indicating transitions from lower levels to higher levels, which are not explained in the paragraph that references Fig. 6c in the text.

Response：Fig. 6c's description is not clear enough. We add more details to explain the traversal process of the 1D min-max binary tree, as described below:

in lines p.319~327

Q6. In Fig. 8, are (a) and (b) switched (or are their descriptions switched in the text)?

Response：Indeed, this is one of our mistakes. The description of the picture does not correspond to the picture. We have switched the text.

Q7. In Table 1, I presume the slashes indicate categories that are not applicable. Perhaps "N/A" would be clearer than just a slash.

Response：Yes, the slashes indicated categories that are not applicable. We use "N/A". We replaced the original slash with N/A.

Q8. The section "Discussion" seems to be too short as a section unto itself.

Response： Yes, We added the following discussion in this chapter, including the type of scattering, smooth transition, number of light sources in lines p.468~478, as shown below:

:

Q9. Overall, the writing is decent, but there are some English mistakes here and there. After editing for content, the manuscript should be proofread again to catch such English mistakes. Some examples are:

p. 1 "slice of disadvantages"

p. 2 "the sampling strategy and steps need to redesign"

p. 2 "And the reasonable..."

p. 2 "And then..."

p. 3 "While further..."

p. 4 "Near clipping plan" and "Far Clipping plan"

p. 6 "visible vator"

p. 6 "by introduced"

p. 6 "density of participating media density"

p. 7 "is taxonomically into three subsets"

p. 7 "two-days flight"

p. 7 "results of Qualitative experiments"

p. 7, "Besides, ..."

p. 9 "mearing distance"

in general, the use of capitalization in the figures

Response：Yes, we corrected the English mistakes you pointed out, and rechecked the full text.

p.1"slice of disadvantages" -> p. "lots of disadvantages"

p. 2 "the sampling strategy and steps need to redesign"->"the sampling step size and sampling strategy need to be redesigned"

p. 2 "And the reasonable..." -> Due to unreasonable logic, the sentence and its context have been rewritten.

p.2 "And then..." -> "then..."

p. 3 "While further..." ->" further..."

p. 4 "Near clipping plan" and "Far Clipping plan"->"near clip plane" and "far clip plane". As described at line X. Algorithm 1.

p. 6 "visible vator"->"visible vector"

p. 6 "by introduced"->"by introducing"

p. 6 "density of participating media density"->" density of participating media "

p. 7 "is taxonomically into three subsets"-> is divided into three subsets

p. 7 "two-days flight"->" Flight track of two days "

p. 7 "results of Qualitative experiments" ->"visual quality "

p. 7, "Besides, ..." ->"We then... "

p. 9 "mearing distance"-> Due to unreasonable logic, the sentence and its context have been rewritten.

Reviewer #2:

Q1. This manuscript describes a real-time software system for rendering atmospheric effects in large-scale outdoor scenes, in the context of a flight simulator. It builds on the existing techniques of Epipolar Sampling. The paper's results appear quite reasonable and look very nice, and the strategies being described seem to be valid approaches to solving the problem. The results are described reasonably and report the performance (timing) measurements that are needed.

However, I would describe the description of the system as rather cursory and incomplete. It would not be possible to reproduce the results from the level of detail that appears in the paper. The precision and completeness with which the method is explained and derived is markedly lower than other work in the area, e.g. refs 12 and 19 and the following paper that is not cited:J. Chen et al. "Real-time volumetric shadows using 1D min-max mipmaps," I3D 2011, doi:10.1145/1944745.1944752

Response：Yes, we noticed the problem of completeness and detailed description. We

made modifications from the following aspects. First, we added another 7 references[35-41] in this field (including J. Chen et al. "Real-time volumetric shadows using 1D min-max mipmaps,"). Second, we discussed which aspects are new and which reuse ideas from prior work(See chapter Overview, p.106~207). Third, we added more algorithm description, such as Algorithm "Ray marching by the improved ES" (see Algorithm 2 Ray marching of the improved ES). Fourth, for each part of the algorithm, whether it is original or modified on the basis of predecessors, we added analysis and contextual descriptions (p. 241~245, p.274~286, p.329~340). Fifth, we uploaded the source code and data set of this manuscript. Finally, we added several experiments compared with the ground-truth references to strengthen the validity and completeness of the verification (p. 411~432).

Q2. The results of the system are not compared to any ground-truth references (e.g. by slow path tracing methods) to show how accurate they are, nor is the accuracy compared to previous methods (only the performance, and only to reference 12).

Because of this it is difficult to authoritatively judge correctness. Though it must be said that in the real time context, correctness in practice often means that it produces good-looking images fast enough, which this system does seem to.

Response： Yes, the results of our system are not compared to any ground-truth references to show how accurate they are. We added three experiments and introduced the experimental results of ground-truth references as a benchmark for comparison. As shown in Fig 11, the performance of volumetric light (including multiple light sources) are tested in variety of scenarios. Each row from left to right then shows the image quality that method A, B and the proposed method can provide while maintaining the same FPS. It can be observed that the method in this paper achieves a more realistic volumetric light quality with minimal antialiasing. As demonstrated in Fig 12, we test how long various of the algorithm take with the same visual quality. Fig 13 shows the density change of the participating media and the

interaction with multiple light sources.

Q3. As I read the expectations of PLOS ONE for data availability, I think the appropriate interpretation for a graphics paper is that the authors release an open source implementation of the method, together with the models required to replicate the key results of the paper. The authors simply state that all required data is in the paper, but I don't think this meets the spirit of making the data available that is needed to evaluate the paper's claims about its method.

Response : Yes, in this submission, we upload the source code and models of the paper.

Q4. The English is not very standard, but I did not have any trouble understanding it, so I would not think this should be a barrier for this manuscript.
On the whole, though it's not obvious to me exactly how PLOS's criteria apply to a computer graphics paper, I think it is safe to say that the paper does not meet the expectations for completeness, validation, and comparison against prior art that would be expected by a journal or peer-reviewed conference in computer graphics. The paper does not clearly state what technology is new and what is adopted from prior art, and it provides no comparisons to illustrate the benefits of the new components introduced in this manuscript. Also, it fails to cite the paper noted above (though it does cite a related one) which seems to overlap closely with the min/max tree methods used here for adaptive integration along epipolar lines.
I have made the recommendation "major revision" because I do believe a publishable paper can be written about this system. It needs to include a complete and clear description of the system and the algorithms it uses, clearly discuss which of those algorithms are new, demonstrate the benefits provided by the new methods, validate the accuracy of the images being produced, and (if my interpretation of the data policy is correct) include code and data that can be used to replicate the results.

Response : For the English description of this article, we have made some

optimizations in this submission. Based on the opinions of the review experts, we have made a lot of modifications to satisfy the completeness, validation, and comparison against prior art that would be expected by a journal or peer-reviewed conference in computer graphics. We adjusted the chapters of introduction, overview and related works to elaborate on the reasons for this article. And we answered why the existing methods cannot meet our needs. It distinguishes in detail which cited previous methods and which parts are original. For the cited methods, we mainly describe our modifications to make it suitable for this application. For the new methods, we explained the main contributions. We cited the papers closely related to this article, discussed the differences carefully, and used their results as an important comparison benchmark for the experiment in this article. We upload the source code and models of the paper in this submission.

Reviewer #3:

Q1. Volumetric lighting is a hard problem in real-time rendering. This submission presents a technique to add it to a flight simulator, which has more rigid performance constraints than many real-time applications. This paper presents the engineering decisions made for a particular implementation, which is a reasonable publishable contribution. However, there are some problems with the submitted work. The first major problem is unclear how the authors want it evaluated. The text suggests it is a novel research algorithm that solves what the authors claim were previously unachievable effects. The video and images suggest it should be evaluated as a simulation that faithfully and accurately reproduces the images see from an airplane cockpit, without worrying about specific complex scattering and shadowing. As a reviewer, I read these differently. The first is a research paper, the second is an engineering / systems paper. Unfortunately, because of this lack of clairty, in the current state, I couldn't recommend it for acceptance in either category.

Response：This method was proposed to solve existing engineering problems. During the research, we refer to many works in this field for solutions. However, the existing

methods cannot directly achieve the desired effect. Through continuous verification and modifications, a new framework was formed. The method in this paper is not a completely general solution for volumetric light. But it still has certain research and application value in large-scale outdoor environment. At the same time, we made a lot of modifications in accordance with the requirements of research paper as follows: a, we added many ground-truth reference in this field as the most important benchmark of discussion and experimental comparison. b, we clearly discussed which aspects are new and which reuse ideas from prior work. c, for each part of the algorithm, whether it is original or modified on the basis of predecessors, we added analysis and contextual descriptions. d, we strengthen the verification of the experimental results by adding 3 more tests, See Figure 11, 12 and 13. After this major revision, I believe the manuscript will be closer to the publication standard of research papers.

Q2. The second major problem is the paper skips citing many closely-related papers. For instance, the work on "Voxelized Shadow Volumes" by Chris Wyman (from High Performance Graphics 2011) and the follow up "Imperfect Voxelized Shadow Volumes" from HPG 2013 use a very similar epipolar space sampling technique that aligns ray marching samples with the alignment of samples in memory, and this scales to many lights (at least in the "imperfect" case). While other work by Chen et al. is cited, the paper "Real-Time Volumetric Shadows using 1D Min-Max Mipmaps" from Symp. on Interactive 3D Graphics 2011 is not cited, and their collective work renders volumetric shadows on rectified shadow maps using 1D mipmaps / binary trees (similar to the approach described in the text). Citing the Tevs et al I3D 2008 paper "Maximum mipmaps for fast, accurate, and scalable dynamic height field rendering" would also be appropriate given the use of 1D trees for traversa

l. Also, it's not clear why a Gaussian blob model was chosen for heterogeneous media. There are a variety of models that can be used for media ranging from Gaussian radial basis functions up to complex simulation-driven formats used in film renderers. I would have hoped to see some discussion for the rationale and citations of the

relevant related work. Certainly, the real-time work typically relies on homogeneous media, but there is more exploration of more complex alternatives in the offline rendering literature. And there are various real-time solutions for more complex scattering functions (e.g., Pegararo et al's work "A closed-form solution to single scattering for general phase functions and light distributions" from Computer Graphics Forum 29(4).)

Response：Yes, we were indeed missing some important reference. We added all the references above in this version of manuscript. We will reply one by one to the similarities and differences newly cited above(p. 127~176).

a. The work "Voxelized Shadow Volumes" by Chris Wyman and the follow up research used a very similar epipolar space sampling technique that aligned ray marching samples with the alignment of samples in memory, and this scaled to many lights. Though the methods decoupled geometric complexity and reduced visibility test cost, its versatility was not as good as shadow map where the output of the shadow map could be shared with many components related to light and shadow. Besides, the VSV (voxelized shadow volumes) had some shortcomings, such as aliasing near singularity and some care in implementation details for robustness (p. 152~161).

b. 1D min-max mipmaps by Chen et al. is classic method in solving the visibility determination of sampling points of ES. However, its rectified shadow map was complicated and existed restrictions on the scope and angle of the view ray (p. 145~151).

c. Similarly, Tevs et al. used 1D trees to achieve fast, accurate and scalable dynamic height field which inspired us by the use of 1D min-max mipmaps. However, it mainly focused on real-time rendering of large-scale height field which was included in our terrain system(p. 87~90)

Q3. The third problem with the paper is the fairly simplistic evaluation. One of the key features of this work, as far as I can understand, is the handling of shadowing and

visibility inside the media. The only image in the paper that clearly shows shadows is Figure 9b. And this is a scene where a radial image-space filter emanating from the sun would give nice results (this is frequently what game developers used for volumetric shadows today). It is important to see how the proposed approach from the paper works in a variety of scenarios and not just fairly simple situations. Additionally, one key feature of the paper is that it applies to not just one light source, but to many light sources. Only one example is provided in the submission (in Figure 11), and as far as I can tell this image does not have any shadows. Essentially: the results are unclear if this algorithm works for providing shadows from many lights. (Or if "shadows" and "many light source" are two independent improvements that do not work together.) Additionally, as far as I can tell, the only change in media density is the fairly simplistic decrease in density with altitude (shown in Figure 9a). It is unclear if the proposed approach in Equations 1, 2, and 3 is evaluated. Certainly, the texture ping-pong / ray-marching approach described in lines 219-223 is probably unnecessary with media density that changes so smoothly. The video isn't particularly useful -- it shows only simplistic relatively homogeneous media, as far as I can tell, without any shadowing, and without any ground truth comparisons. Ideally, for a flight simulator, you could compare with actual photos in similar situations.

Response：Indeed, the existing experiments did not take into account the in a variety of scenarios and not just fairly simple situations. It also failed to show the characteristics of supporting multiple light sources. Additionally, it was impossible to distinguish the interaction between variable density of participating media and volumetric light. To solve the problems above, we added three experiments. As shown in Fig 11, the performance of volumetric light (including multiple light sources) are tested in variety of scenarios. Each row from left to right then shows the image quality that method A, B and the proposed method can provide while maintaining the same FPS. It can be observed that the method in this paper achieves a more realistic volumetric light quality with minimal antialiasing. As demonstrated in Fig 12, we test how long various of the algorithm take with the same visual quality. Fig 13 shows the

density change of the participating media and the interaction with multiple light sources (p. 411~432).

Q4. A fourth problem with this submission is it skips the most important aspect of a research paper (in my opinion): describing why the work is needed. Typically, one would use a known, existing algorithm unless it does not solve a problem required by your application (i.e., your flight simulator). There is no need to develop a new algorithm that may have new and unknown problems without a good reason. These reasons should be clearly specified, and the choices made by a novel algorithm should derive from the functionality desired. This paper does not tie together the algorithm with the rationale, it simply specifies the algorithmic steps in a bulleted list without describing why these choices are better than any possible alternatives. Once you motivate each step in the new algorithm, it is much easier to judge whether the proposed approach will significantly improve over prior art. (Also, it often makes it easier to design evaluation images that clearly show the improvement.)

Response：We adjusted the chapters of introduction, overview and related works to elaborate on the reasons for this article(p.2~207). And we answered why the existing methods cannot meet our needs. It distinguishes in detail which cited previous methods and which parts were original. For the cited methods, we mainly describe our modifications to make it suitable for this application. For the new methods, we explained why it was better than other possible alternatives and the main contributions of proposed method. For each part of the algorithm, we analyzed and described context based on basic principles. For the core algorithm, we added details for easier judgement of performance and evaluation. Further, we redesign the evaluation images that clearly show the improvement by adding three new tests, See Figure 11, 12, 13 (p. 411~432).

.

Q5. As far as clarity of the text, I would provide the following suggestions:

1) Provide an overview section that summarizes the new approach and why this new approach addresses the failures of prior work

2) Revisit the enumerated contributions (currently on lines 47-60). Make sure each one is measurable. Make sure these measurements are provided in the evaluation.

3) For each algorithmic section, provide some reminder of what the next step in the algorithm needs to solve, and discuss which aspects are new and which reuse ideas from prior work.

Response：Yes, we provide an overview section(p. 106~207) summarizes the new approach and why this new approach addresses the failures of prior work. We rewrite the enumerated contributions (currently on lines 33-48). Each contribution was measurable and supported by experimental results. For each algorithmic section, we remaindered the problem need to solve (p. 241~245, p.274~286, p.329~340). We then discussed which aspects are new and its reason and which reuse ideas (p.177~207) from prior work (including adaptive improvements for applications).

--End of Reply---

Once again, thank you very much for your constructive comments and suggestions which would help us both in English and in depth to improve the quality of the paper.

Kind regards,

TanShihan

E-mail: 530916232@qq.com
