# Supplementary material for: Adaptive volumetric light and atmospheric scattering
Source: PLoS One. 2020 Nov 18;15(11):e0242265. doi: 10.1371/journal.pone.0242265 (PMC7673549; doi:10.1371/journal.pone.0242265)
Supplement: Attachment — Submitted filename: Response to Reviewers.pdf [file pone.0242265.s007.pdf]

Cover Letter

Dear Editor and reviewers

Thank you very much for giving us an opportunity to revise our manuscript. We appreciate the editor and reviewers very much for their constructive comments and suggestions on our manuscript entitled "Adaptive volumetric light and atmospheric scattering" (ID: PONE-D-20-19144R1).

We have studied reviewers' comments carefully. According to the reviewers' detailed suggestions, we have made a careful revision on the original manuscript. All revised portions are marked in highlight in the revised manuscript which we would like to submit for your kind consideration.

Kind regards.

TanShihan
E-mail: 530916232@qq.com

Rebuttal Letter

Dear Editor and reviewers:

Thank you for your letter and the reviewers' comments on our manuscript entitled "Adaptive volumetric light and atmospheric scattering" (ID: PONE-D-20-19144R1).

Those comments are very helpful for revising and improving our paper, as well as the important guiding significance to other research. We have studied the comments carefully and made corrections which we hope meet with approval. The main corrections are in the manuscript and the responds to the reviewers' comments are as follows (the replies are highlighted in blue).

Replies to the reviewers' comments:

Reviewer #1:

Q1. This is a revision of manuscript PONE-D-20-19144. From the reviews of manuscript PONE-D-20-19144, the reviewers, including myself, made suggestions that were based mostly on properly framing the work that the authors' have completed in comparison to previous work, providing more and clearer detail regarding the authors' algorithm (including providing the source code itself), and providing more demonstrations of the success of the authors' algorithm. The authors have made numerous changes to the manuscript in response to those suggestions. In the revised manuscript, the reader now has a better perspective on the novelty of the authors' work in comparison to previous work, and some important references to previous work that were missing in the original submission have now been included. The subsections titled "Related works" and "Overview" are a little unconventional, and the Introduction could have been structured differently while including the same information, but the necessary content is now there. The description of the method is now more detailed and clearer. The results from the previous submission looked good, and the additional results that the authors have included in the current submission, including comparisons to ground-truth references, also look good.

I think the paper could still use some minor revisions in terms of editing for English, especially in the newly added sections. Just a couple of examples include:

(1) The use of the word “finally” is incorrect in a number of places in the text.

(2) There are mistakes in capitalization, such as “chen” on line 277, “Transversed” on line 285, and “Textures” on line 357.

Response：(1) We have revised the use of conjunctions, including but not limited to “finally”, see the highlighted part for details.

(2) We have modified these mistakes in capitalization, such as “chen” ->”Chen” on line 246, “Traversed”->” traversed” on 255 and “Textures”->”texture” on line 333.

Reviewer #2:

Q1. This revision is commendably responsive to the reviews. The manuscript is much improved in terms of detailed description of the contributions and proposed methods, and in evaluation of the results of those methods. Good discussion of the contributions of previous work relative to the current paper is included, and detailed pseudocode is provided to clarify the details for those who wish to implement the method. For me the level of novelty and the improvements in performance shown here are sufficient for a publication, and subject to the caveats below the paper appears to be near the right condition for a minor-revisions accept. But some of the below involve enough uncertainty that I'm still at "major revision".

* The source of the implementations of Chen et al. and Ali et al. is not mentioned. It should be clarified that it's the authors' own reimplementation (if it is), any differences with exactly what was proposed in those papers should be described, and these reimplementations should be included in the released source code.

Response： Unfortunately, the source code of Chen et al. is not found. We re-implement it on basis of other 1D min/max mipmaps source code on “gethub”, and modified it step by step according to the original paper. For the approach by Ali et al.,

we find its implement. The source code of the above two articles have been added to this submission.

Q2. * The source code contains comments in Chinese, many of which seem to be translations of the English but some of which seem to include new information; for publication everything should be in English.

Response：Yes, the Chinese description in the source code has been changed to English for publication, see the new version source code for details.

Q3. * The text mentions a compute shader which I do not see in the source code.

Response： Because in the historical version, some components do not support the compute shader, we put all this type files in a separate folder, resulting in the missing of the compute shader part in the last version. This submission will include this one.

Q4. * The understandability of the English has become worse in this revision, and I think it now needs some work to bring it up to the standard of "intelligible" everywhere. Really the paper should get a thorough edit by someone with more experience in written English.

Response： Yes, an American student in this field was invited to standardize and improve the language description, see the highlighted part for details.

Q5. It's worth pointing out that the source code does not meet the standard of replicability, since it cannot be compiled without the rest of the system from which it comes. But to the extent it contains the full implementation of the proposed method, I think it does meet the standard of revealing exactly what was used to compute the results. Including the implementations of the competing methods would make this more complete.

Response： Yes, we add the implementations of the competing methods in the source-

code file, such as soft bilateral filtering shadows by Ali HH et al. and 1D min-max mipmaps approach by Chen et al.

Q6. * The timing results appear to be from the same experiments as before, but with the methods relabeled. It's good that they are now labeled in a way that credits the pieces to earlier papers, but I am a little concerned that the Chen et al and Ali et al papers, which were not previously referenced, are now the labels for results that were included before. Were these methods implemented before but not cited? Or is the implementation that was made without referencing these papers now being used to represent those methods in the comparison? Both interpretations are a problematic.

Response：I’m sorry that the previous description is not clear enough. In the previous version of the manuscript, the experimental comparison was divided into several categories according to the methods, without specifying which paper was used as the reference. Indeed, for the “ES+BT” type, the paper we actually referred to at the time was “Chen J, Baran I, Durand F, Jarosz W. Rendering images with volumetric shadows using rectified height maps for independence in processing camera rays; 2016.” (Number 28 in the references in our manuscript) This paper is the subsequent version of “Real-time volumetric shadows using 1D min-max mipmaps” by the same author in which ES and 1D min-max acceleration data structure for rectified shadow map is also the core algorithm. Unfortunately, the source code of these two paper is not found. We re-implement it on basis of other 1D min/max mipmaps source code on gethub, and modified it step by step according to the original paper. Since the implementation of the two versions of the experimental reference paper is essentially the same version we implemented, only the name has been modified, and the results of the experiment remain unchanged. Similarly, the approach by Ali et al. was called “ES+SS” in the previous version of manuscript, which is actually the same work by Ali et al in this version. Therefore, the experimental results have not changed, and the source code will be submitted in this version.

Q7. * The paper by Ali et al. (formerly just called "subsampling" without any explanation) is included in the comparison but not discussed at all in the prior work. It also seems to produce the closest results to the new method. The differences between and relative merits of the methods need to be discussed.

Response：Yes we discussed the paper by Ali et al. as follows: This study introduces the soft bilateral filtering shadows method of image-based shadows. Its main contribution is to obtain soft shadow with the lowest number of samples and the

image quality is compensated by bilateral filtering. Compared with the proposed approach, the first and foremost, both methods achieve a good balance between efficiency and image quality. This the why the method is very close to the proposed approach. However, this method reduces the samples for efficiency, which leads to sparse distribution of ray marching, where some unnatural light beams will appear. See the highlighted part for details on line 408-414.

Q8. * The authors have borrowed two complete sentences of text from the reviews and incorporated them into the discussion of prior work: "...a very similar epipolar space sampling technique that aligns ray marching samples with the alignment of samples in memory, and this scales to many lights..." and "There are a variety of models that can be used for media ranging from Gaussian radial basis functions up to complex simulation-driven formats used in film renderers...". This text is not the authors' work; reviewers are not here to write papers for authors. I'm not sure what PLOS's policies say about this.

Response：Yes, we have re-modified the related description.

"...a very similar epipolar space sampling technique that aligns ray marching samples with the alignment of samples in memory, and this scales to many lights..."

to "… a very similar approach by using epipolar space sampling, which aligns the samples of ray marching in memory. Then, many lights are supported by their subsequent works" on line 135-137.

"There are a variety of models that can be used for media ranging from Gaussian radial basis functions up to complex simulation-driven formats used in film renderers..."

to" There are various methods to generate air media, from Gaussian blur to physically driven method used in offline renderers" on line 302-303.

--End of Reply---

Once again, thank you very much for your constructive comments and suggestions which would help us both in English and in depth to improve the quality of the paper.

Kind regards,

TanShihan

E-mail: 530916232@qq.com
